# Adherence to treatment guidelines for uncomplicated malaria at two public health facilities in Nigeria; Implications for the ‘test and treat’ policy of malaria case management

**DOI:** 10.1186/2052-3211-7-15

**Published:** 2014-11-14

**Authors:** Charles C Ezenduka, Mathew J Okonta, Charles O Esimone

**Affiliations:** Department of Clinical Pharmacy & Pharmacy Management, Faculty of Pharmaceutical Sciences, Nnamdi Azikiwe University Awka Agulu campus, Agulu, Nigeria; Department of Clinical Pharmacy & Pharmacy Management, Faculty of Pharmaceutical Sciences, University of Nigeria, Nsukka, Nigeria; Department of Pharmaceutical Microbiology & Biotechnology, Faculty of Pharmaceutical Sciences, Nnamdi Azikiwe University Awka, Agulu campus, Agulu, Nigeria

**Keywords:** Uncomplicated malaria, Treatment guidelines, Policy adherence, Antimalarial drugs, Prescription practices, Health facility, Nigeria

## Abstract

**Objectives:**

Adherence to treatment guidelines for uncomplicated malaria is critical to the success of malaria case management. Poor adherence has implications for increased malaria burden, in view of the risk of widespread parasite resistance and treatment failures. This study analyzed the diagnostic and prescription pattern for uncomplicated malaria at two public health facilities, south east Nigeria, to assess the current state of compliance to policy guidelines on the use of artemisinin-based combination therapy (ACT).

**Methods:**

Retrospective audit of patients’ records, treated for uncomplicated malaria, between the months of January and March 2013, was undertaken at two public health facilities. Demographics, diagnostic information, medication and cost data were extracted. Questionnaires were distributed to providers to assess their malaria treatment intent. Data from the facilities were analyzed and compared for similarities and systematic differences, and conformity to malaria treatment policy, in terms of laboratory diagnosis, use of ACT, co-medication and cost of medication.

**Results:**

A total of 2,171 records of patients who had been treated for uncomplicated malaria were analyzed. Of these, 1066 (49%) were sent for laboratory confirmation of malaria using mostly microscopy, out of which 480 (45%) tested positive. 51% (1105) of the prescriptions was on the basis of presumptive treatment. 58% of slide negative results received antimalarial drugs. 93% of patients received ACT, with artemether-lumefantrin, AL (50.5%) as the most prescribed antimalarial drug. Monotherapy accounted for 7% of prescriptions, comprising mostly sulphadoxine + pyrimethamine, SP (46.5%) and monotherapy artemisinin, AS (29.2%). 97% of the prescriptions received at least one co-medication. Antibiotics were prescribed to 50% of patients. Overall, median cost of medication was N1160.00 (US$7.48 (US$0.19 - 267.87) per case, higher in tertiary than the secondary facility. There were significant variations in treatment practices between the two facilities.

**Conclusion:**

Evidence suggests good compliance to policy on the use of ACT as first line treatment for uncomplicated malaria. However, there exists significant scope for improved diagnosis and rational drug use, to enhance accuracy of treatment, reduced wastages and risks of adverse drug reactions, in line with the goals of ‘test and treat’ policy of malaria case management.

## Introduction

Case management remains a major strategy for effective control of malaria, comprising diagnosis and prompt treatment with effective antimalarial drugs. In recognition of its high efficacy and potential for preventing development of parasite, ACT was recommended for the first line treatment for uncomplicated malaria in 2001 [1]. However, in view of the need to achieve efficiency in malaria treatment and enhance the goals of case management, the World Health Organisation (WHO) recommended the test, treat and track (TTT) policy, which emphasises improved diagnosis of malaria infection, prompt treatment with effective antimalarial drugs and regular monitoring through routine information system to ensure effective implementation [2, 3]. Subsequently many malaria endemic countries in Africa adopted the policy, highlighting the importance of parasitological diagnosis using either microscopy or rapid diagnostic tests (RDTs) for malaria treatment, in all age groups and in all epidemiological settings [3, 4]. This led to an increased supply of RDT. However, major challenge to implementation of policy guidelines has remained that change in policy recommendations does not always translate to immediate and effective change at health care provider levels and hence, inadequate quality case management at the point of care [5]. Consequently, inappropriate practices in the provision of malaria treatment have been reported among healthcare providers, from facilities in many malaria settings [5–8]. This constitutes non-adherence to treatment guidelines, contributing to undermining the goals of malaria treatment policy.

Adherence to policy guidelines by healthcare providers and patients is essential for achieving the success of this policy [3, 9]. Studies in developing countries suggest that many years after the introduction of ACT, inappropriate prescription and use of antimalarial drugs persist, at both public and private health facilities [6–9]. With better exposure to information, it is expected that health workers in public facilities would have better access to malaria treatment guidelines, and be more likely to adhere to recommended strategy for uncomplicated malaria [7]. Although the public health facilities, more than the private sector, are known to largely conform to policy on the use of antimalarial drugs [10, 11], reports indicate substantial inappropriate treatment practices, such as presumptive treatment, treatment of slide -negative results, co-medication (poly-pharmacy), use of low quality and expensive ACT and monotherapy [7–9]. These lead to wastages and inefficiency in the implementation of malaria case management, thereby increasing the risk of widespread resistance and treatment failures.

A study in Kenya [12] demonstrated how lack of adherence to treatment guidelines was associated with inappropriate prescription practices. The use of sub-therapeutic doses of drugs contributes to the risk of developing parasite resistance [13]. Drug regimens with long duration of treatment, such as monotherapy artesunate also contribute to poor adherence [8, 14]. Concomitant medications further contribute to inappropriate prescription through polypharmacy, which increases risks of drug interactions, adverse drug reactions, non-adherence and treatment failures, in addition to high cost of care [11]. Presumptive treatment in health facilities have been shown to be widespread even with the availability of diagnostic instruments. Evidence also suggests frequent use of antimalarial drugs by health workers on slide-negative results [3, 5, 7, 15]. Given the substantial misdiagnosis of febrile patients for malaria cases, using presumptive diagnosis, the use of antimalarial drugs under the current policy involving the use of more expensive ACT, represents substantial economic losses.

Several factors have been identified to be responsible for the non-adherence of prescribers to recommended guidelines. Inadequate supply of recommended drugs, unnecessary use of more expensive recommended drugs, continuous availability of monotherapy, staff shortages and high work load, as well as contradicting training messages that confuse workers [9, 16], have been reported. These represent supply-side factors which limit effective implementation of malaria treatment policy. In Nigeria, although change in policy to the use of ACT was introduced in 2005 [17], inappropriate practices have been reported by many studies [7, 10]. Understanding these issues is essential for generating information for implementing strategies to improve effective malaria treatment. This study was aimed to describe and assess the diagnostic and treatment patterns for uncomplicated malaria at two public health facilities in Nigeria, and determine current conformity to policy guidelines.

## Methods

### Study population

The study was undertaken in Anambra state, south-east Nigeria, with a total population of 4.18 million inhabitants by 2006 Nigerian census, considered as the second most densely populated state in the country (1,500 – 2000 persons per km^2^). Divided into three senatorial zones, the state has 21Local government Areas (LGAs). The people, who are predominantly ethnic Ibos, are involved in farming as the main occupation, while a significant number is into trading and commerce. Malaria transmission in the state is perennial with incidence rate of between 10 – 35% and peak season coinciding with the rainy season, running between March and October every year. Children and pregnant women are the most affected by malaria. There are about 382 primary health centres (PHCs), managed by the LGAs, 32 secondary health facilities run by state government and two tertiary health facilities owned by the state and federal governments respectively. As in most of the Nigerian population, *Plasmodium falciparum* is the dominant malaria specie in the state, and artemether-lumefantrine (AL) is first-line drug for the treatment of uncomplicated malaria since 2005, followed by Artesunate-amodiaquine (AA) as alternative first line drug. Presently, a wide range of ACT is registered in Nigeria for the first line treatment of uncomplicated malaria, such as dihydroartemisinin-piperaquine (DHAPQ) and artesunate- mefloquine (ASMQ) [11]. The state is one of the most important sources of drugs supply in Africa, due to the presence of the popular ‘Bridge-Head’ market, a known center of drug trade, located in Onitsha, the largest commercial city in the state.

### Study sites and setting

The study was carried out in two sites of a federal institution, the Nnamdi Azikiwe University (NAU). These include, NAU Medical Center (NAUMC) located at Awka, the state capital and NAU Teaching Hospital (NAUTH), Nnewi, the second largest commercial city in the state. The sites represent primary/secondary and tertiary healthcare facilities involved in malaria treatment, and were selected because of the opportunities they present for collecting quality and reliable data.

The NAU Medical Center is a healthcare facility which primarily provides outpatient services in a university community of about 50,000 people, as the catchment population. Significant number of the Awka community also accesses care at the facility. It has about 10 medical officers who provide clinical services to patients in addition to nurses, pharmacists, laboratory officers and other health workers. The center has a functional laboratory which provides microscopy and RDT services. There are 10 in-patient beds, which are used to provide brief admissions for emergency cases. Over 10,000 outpatient cases are treated annually at the facility. The supply of anti-malarial drugs is carried out through a process that is based on a procurement guideline. Donors also provide support through donations of drugs, such as the Affordable Medicine Facility-malaria (AMFm) drugs, though quantities of supply are relatively small. Availability of antimalarial drugs in the facility is said to be regular although, in many occasions there is a limited range of the products at any one point in time, due to purchasing procedures. Payments are made by all patients including staff, students and community members, who access services at the center. Payment by the students is deducted from fees paid in advance.

NAUTH is a 500-bed tertiary healthcare facility providing a variety of specialized clinical and teaching services. It is the main referral public health facility in the state run by the federal government. As at 2010, the hospital had total staff strength of about 2400 workers, spread across the various clinical and non- clinical departments; comprising over 300 doctors, 400 nurses and 62 pharmacists, including intern pharmacists. There are 15 wards with estimated 80% bed occupancy. The general outpatient department (GOPD) attends to over 12,000 out-patient visits annually. Patients pay for services and their drugs at the point of delivery.

### Study design and data collection

A descriptive cross-sectional study was conducted, based on retrospective cohort event monitoring of patients treated for uncomplicated malaria in the course of medical practice. Facilities were selected to ensure availability of adequate patient load and coverage, and the need to recruit large enough patients in a short period. Hospital records of patients diagnosed or treated for uncomplicated malaria, within a three month period of between January and June 2013, were collected and audited. Cases of severe/complicated malaria and pregnant women were excluded. Two pharmacy graduates were trained to extract and record the data from the patients’ records into a pre- designed Excel data form. Individual patient-level records and prescription were collected for each outpatient treated at the facilities. Collected data included demographics, diagnosis, laboratory tests results, drugs prescribed, number of drugs, cost of drug prescription and co-prescribed medications, over the study period. Medication doses and route of administration were not documented, but the drugs were prescribed and dispensed in age-related doses. Pre-tested semi-structured questionnaires of 15 questions were distributed to the prescribing physicians in selected facilities, to assess their prescribing intent in the treatment of uncomplicated malaria, in terms of the use of laboratory services, antimalarial drug-use and prescription pattern.

### Data management and analysis

Data was double entered, cleaned and managed with Excel spreadsheet. Analysis was carried out for diagnostic approaches, (use of microscopy and/or RDT), use of ACT, monotherapy, concomitant medication and cost of medication. Prescriptions were categorised into ACT and mono therapy. Analysis was carried out at whole facility level (facilities are located in the same state) and then separately for individual facility, to assess the differences in treatment pattern between the facilities in conforming to malaria treatment guidelines. Conformity to treatment guidelines was on the basis of laboratory diagnosis, use of ACT and rational use of drugs.

Data were collected using Excel spreadsheet and statistical analysis was performed using SPSS version 16 (SPSS Inc., Chicago, IL, USA) and GraphPad Prism 5 for Windows, (GraphPad Software, San Diego California USA). Association between variables of interest and the prescription of antimalarial drugs were estimated using logistic regression. Chi squared test of independence was used to determine association between categorical variables, independent student’s *t*-test for continuous variables, and univariate analysis to predict the prescription of ACT and concomitant medications. Statistical significance was set at p = 0.05.

### Ethical considerations

The study obtained approval from the NAUTH Ethical Committee as part of a larger study on the pharmacoeconomics of malaria treatment in Nigeria. Each doctor’s consent was obtained to participate in the questionnaire survey. The study did not involve direct patient contact hence patient consent was not necessary.

## Results

### Study characteristics and malaria diagnosis

**Figure 1:**
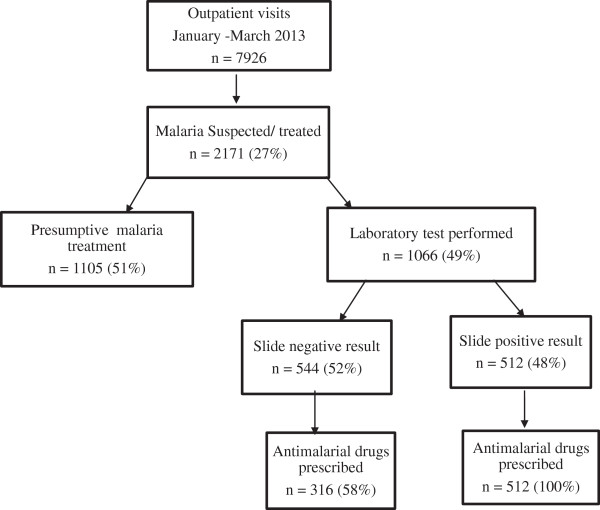
**Schematic summary of selected malaria cases included in the study.**

A total of 7949 outpatient visits were identified, out of which 2171(27.3%) cases were treated for uncomplicated malaria. Ninety-four (4%) records were excluded due to either severe malaria or incomplete/missing information on variables of interest. 49% (1066) of those treated for malaria was sent for laboratory examination while the rest, 51% (1105) was based on presumptive diagnosis. 48% of laboratory diagnosis tested slide positive while 52% tested negative. Only the tertiary health facility reported the use of RDT. Figure 1 shows the graphic presentation of selection process. The proportion of uncomplicated malaria cases was higher at the medical center, 47% (1261/2674) compared to the teaching hospital, 17% (910/5252).

Characteristics of cases finally analyzed (Table 1) shows that overall, females (56%) outnumbered males (43%). Gender disparity was higher at the teaching hospital than the medical center where females, 65% outnumbered males, 35%, (p <0.0001). Most cases (62%) fall between 19 years and above, at a median age of 23 years, ranging from one month to 98 years. Children under 5 years accounted for 10% of total cases. The proportion of children under five years was higher at the teaching hospital, 20% (181/910) compared to the medical center, 2.6% (33/1261). The proportion of ACT prescribed at the medical center (95%) was significantly higher than the teaching hospital, (91%, p < 0.003).

**Table 1:** **Characteristics of study population by facility**

Variable	Total facility n (%)	NAUMC Awka n (%)	NAUTH Nnewi n (%)	Chi square difference	p-value
Total number of outpatients visits	7926	2674	5252	-	-
Proportion of malaria cases	2171 (27)	1261 (47)	910 (17)	729.8	0.0001***
Mode of diagnosis					
Microscopy	939 (43)	650 (52)	288 (32)	9.95	0.0016**
RDT	128 (6)	-	128 (14)	-	-
Presumptive	1105 (51)	611 (48)	494 (54)	0.875	0.3497
Gender					
Female	1207 (56)	635 (50.4)	573 (63.0)	42.09	0.0001***
Male	942 (43)	626 (49.6)	316 (34.7)	-	-
Unknown	22 (1.0)	-	21 (2.3)	-	-
Age category (yrs)				20.29	0.0001***
Under 5	214 (10)	33 (2.6)	181 (20)	-	-
5 – 12	52 (2)	22 (1.7)	30 (3)	-	-
13 – 18	104 (5)	78 (6.2)	36 (3)	-	-
19 and above	1354 (62)	1026 (81.4)	330 (36)	-	-
Unknown	447 (21)	102 (8.0)	343 (38)	-	-
Median age of cohort (range)	23 (0.1 – 98)	23 (1 – 82)	24 (0.1 – 98)		
Antimalarial drugs					
ACT	2027 (93)	1198 (95)	828 (91)	13.02	0.0003***
Monotherapy	144 (7)	63 (5)	81 (9)	-	-

**= significant: ***= very significant: ****= highly significant.

### Antimalarial drugs prescription pattern

Table 2 shows the distribution of antimalarial drugs (including co-medication) prescribed by facility. A total of 2,171 drug encounters were analyzed. Of these, 93% (2027) contained an ACT, while 7% (144/2171) was prescribed monotherapy. There were some variations in prescription pattern between the facilities. The proportion of patients who received ACT compared to monotherapy at the medical center, 95% (1198/1262) was significantly higher than the proportion prescribed at the teaching hospital at 91% (828/910, p = 0.003). Overall, the pattern of prescription shows that AL, at 51% (1024/2027) was the most prescribed ACT at both health facilities, followed by DHAPQ, 17% (339/2027) and ASAQ 12%. While this pattern was similar at the medical center, it differed from the teaching hospital where artesunate-mefloquine (ASMF) and artesunate-sulphadoxine + pyrimethamine (ASSP) were the second and third most prescribed ACT respectively, after AL. Highest proportion of monotherapy was prescribed as SP, 47% (67/144), followed by artesunate monotherapy (AS), 29.2%. The pattern, however reversed at the teaching hospital where AS, 43% (35/81) was the most prescribed monotherapy, followed by SP, 22% (18/81). More monotherapy (9%) was prescribed at the teaching hospital compared to the medical center (5%). Prescription pattern also varied by age and gender. Higher proportion of SP, 49% compared to other monotherapy agents was prescribed to females than males, 44%. Similarly more females, 29% received monotherapy AS than males, 27%.

**Table 2:** **Distribution of antimalarial drugs and co-medication prescribed by facility**

Antimalarial drugs	Total facility n (%)	NAUMC Awka n (%)	NAUTH Nnewi n (%)	Chi square difference	P = value
Artemisinin-based combinations (ACTs)	2027 (93)	1198 (95)	829 (91)	13.02	0.0003***
Artemether-lumefantrin	1024 (50.5)	647 (54)	377 (45.5)	-	-
Artesunate-amodiaquine	244 (12)	206 (17.2)	38 (4.5)	-	-
Artesunate-mefloquine	232 (11.5)	-	232 (28)	-	-
Artesunate-pyridoxine + pyrimethamine	188 (9.3)	56 (4.7)	132 (16)	-	-
Dihydroartemisinin-piperaquine	339 (16.7)	289 (24.1)	50 (6)	-	-
Mono-therapy	144 (7)	63 (5)	81 (9)	13.02	0.0003***
Artesunate	42 (29.2)	7 (11.1)	35 (43.2)	-	-
Amodiaquine	14 (9.7)	-	14 (17.3)	-	-
Proguanil	15 (10.4)	1 (1.6)	14 (17.3)	-	-
Quinine	6 (4.2)	6 (9.5)	-	-	-
Sulphadoxine + pyrimethamine	67 (46.5)	49 (77.8)	18 (22.2)	-	-
Proportion of co-medication n (%)	1722 (97)	1248 (99)	864 (95)	32.37	0.0001***
Analgesics	1722 (79)	1124 (89)	598 (66)	176.8	0.0001***
Vitamin preparations	1364 (63)	993 (79)	371 (41)	324.8	0.0001***
Antibiotics	1084 (50)	721 (57)	363 (40)	63.18	0.0001***

***= very significant.

### Co-prescribed medication

97% of the patients received at least one co-prescribed medication, at an average of 4 (±1.5) drugs per prescription. Analgesics were the most commonly prescribed co-medication given to 79% (1722/2171) of the cohort, followed by vitamin preparations (63%), and antibiotics (50%). The pattern varied significantly between the facilities and across categories (Tables 2 and 3). Proportion of co-prescribed medications was higher at the medical center, 99% compared to the teaching hospital, (95%, p < 0.0001) (Table 2). This was similar for the most commonly co-prescribed medications at the facilities. Majority, 57% of children under 5 years were more likely to be prescribed antibiotics compared to children between 5 and 12 years (37%, p <0.001) and adults, (53%; p < 0.05) (Table 3).

**Table 3:** **Utilisation of antimalarial drugs across demographic categories**

	Gender		Age category (years)			
Antimalarial drugs	Female n (%)	Male n (%)	Under 5 n (%)	5 – 12 n (%)	13 – 18 n (%)	19 and above n (%)
**Artemisinin-based combination therapy (ACT)**	1127 (93)	881(94)	182(85)	45 (87)	101(97)	1286 (95)
Artemether-lumefantrin (AL)	568 (50)	446 (51)	128 (70)	31 (69)	50 (50)	624 (49)
Artesunate-amodiaquine (ASAQ)	113 (10)	125 (14)	29 (16)	3 (7)	15 (15)	184 (14)
Artesunate-mefloquine (ASMF)	168 (15)	64 (7)	-	-	5 (5)	113 (9)
Artesunate-sulphadoxine + pyrimethamine (ASSP)	115 (10)	72 (8)	18 (10)	11 (24)	9 (9)	82 (6)
Dihydroartemisinin-piperaquine (DHAPQ)	163 (14)	174 (20)	7 (4)	-	22 (22)	283 (22)
**Monotherapy**	80(7)	61(6)	32(15)	7 (13)	3 (3)	70 (5)
Sulphadoxine + pyrimethamine (SP)	39 (49)	28 (44)	4 (13)	2 (29)	2 (67)	44 (63)
Artesunate (AS)	23 (29)	17 (27)	11 (34)	1 (14)	1 (33)	19 (27)
Amodiaquine (AQ)	7 (9)	6 (9)	12 (38)	1 (14)	-	-
Quinine (QN)	2 (3)	4 (6)	-	1(14)	-	5 (7)
Proguanil (PG)	9(11)	6(14)	5 (16)	2 (29)	-	2 (3)
**Co-medication**						
Analgesics	920 (76)	783 (83)	119 (59)	34 (54)	87 (84)	1148 (85)
Vitamin preparations	701 (56)	646 (69)	106 (52)	35 (56)	69 (66)	951 (70)
Antibiotics	569 (47)	502 (53)	115 (57)	23 (37)	59 (57)	720 (53)

NAUMC = Nnamdi Azikiwe University Medical Center, NAUTH = Nnamdi Azikiwe University Teaching Hospital.

### Prescription pattern in children under 5 years

Of the 214 under 5-year old children in this study, 85% (182) received ACT. AL, 70% (128/182) was the most preferred antimalarial drug of choice, followed by artesunate-amodiaquine (ASAQ), 16%. The use of monotherapy occurred most in this group compared to other age categories, and amodiaquine, 38% (12/32) was the most commonly prescribed monotherapy, followed by monotherapy artesunate (AS), 34%. Majority, 57% of the under-5 year old children were more likely to be co-prescribed antibiotics than children between 5 and 12 years (37%, p <0.001) and adults, (53%; p < 0.05) (Table 3). In many cases, the antibiotics were prescribed in combination with cough medications. Co-prescription with analgesics and vitamin preparations was also common in children.

### Costs of medication

Table 4 shows that overall, the median cost of medication (including co-medication) per patient at the two facilities was N1160 (US$7.48) . Medication cost of treatment per patient at the tertiary hospital, N1378 (US$8.89) is about 27% higher than at medical center N1083 (US$6.99).

**Table 4:** **Medication cost of treatment**

	Treatment cost (Naira)			
Variable	Median	Range	Mean	95% CI
**Antimalarial drugs**				
ACT	1176	45 – 41520	1587	1511 – 1663
Mono-therapy	750	30 – 8485	1158	927 – 1389
**Gender**				
Male	1158	72 – 41520	1340	1220 – 1460
Female	1110	30 -16010	1499	1403 – 1594
**Age group**				
Under 5	1062	45 – 7760	1151	1024 – 1277
5 - 12 yrs	776	30 – 3734	914	741- 1087.2
13 – 18	1118	150 – 7900	1243	1067 – 1418
19 and above	1160	85 41520	1503	1412 – 2595
**Facility**	1160	30 – 41520	1559	1486 – 1632
NAUMC	1085	85 – 8000	1171	1135 – 1207
NAUTH	1378	30 – 41520	2101	1940 – 2262

Between the groups, median cost of medication was lowest in children 5 – 12 years, N776 (US$5.01), compared to adults 19 years and above, N1160 (US$7.83). The cost of treatment with ACT, N1176 (US$7.59) is about 1.6 times higher than the median cost of treatment with monotherapy, N750 (US$4.84). It was not possible separating the cost of malaria component of treatment from that of co-morbidity due to inadequate documentation on diagnosis. Hence, medication cost includes the cost of co-morbidity.

Questionnaire distribution recorded 100% response. While every respondent would often request for laboratory test for malaria before treatment, they would also sometimes treat by clinical diagnosis alone. Some of the reasons given for presumptive diagnosis include, confidence in their ability to diagnose malaria without laboratory test (100%), severity of symptom (80%), patient load and lack of waiting time (60%), and previous experience with particular symptom/s (60%). All prescribers (100%) would sometimes prescribe antimalarial drugs in slide-negative results for a variety of reasons; need to prevent malaria infection (40%), unreliable result (60%), and unaware of result (40%). All respondents were aware of availability of malaria treatment guidelines and all would use AL as the preferred ACT of choice for its efficacy and minimum side effects. All doctors use ACT based on recommended guidelines.

## Discussion

This study illustrates practical realities in the treatment of uncomplicated malaria in public health facilities in Nigeria, which has implications for the implementation of malaria treatment guidelines. Findings reflect practices as they relate to the test and treat policy of malaria control. The predominance of the female gender is consistent with many studies [7, 8, 18, 19], where females outnumbered males in health facilities compared to the retail sector. This observation agrees with the suggestion that females make more use of public health facilities than males, who tend to prefer medicine outlets [11]. The finding also implies that women suffer more from malaria attack than males in the study area. The higher proportion of malaria cases at the medical center, (a primary/secondary health facility) is easily explained by the fact that primary health facilities are the main sources of treatment for uncomplicated malaria [9]. The 27% malaria incidence in this study would suggest a declining incidence compared to previous reports of 60% incidence for outpatients consultations in Nigeria [20].

The use of laboratory diagnosis for malaria treatment in the two facilities was limited to 49%, relying substantially on presumptive diagnosis, contrary to the test and treat recommendations of current guidelines. This indicates high incidence of over-diagnosis and over-use of antimalarial drugs, in view of the degree of inaccuracy associated with presumptive malaria treatment [15, 21, 22]. The finding corroborates previous studies in Nigeria and other African countries which have reported widespread limited use of laboratory diagnosis in malaria treatment, even with the presence of diagnostic tools [3, 8, 15, 21]. It reflects the level of confidence and popularity to which prescribers attach to presumptive malaria treatment, as was confirmed by doctors’ responses. This is consistent with findings by Onwujekwe et al. in 2009 and Uzochukwu et al., in 2010, in which over 80% of providers at both hospital and non-hospital alike, are confident in clinical diagnosis of malaria [10, 21]. The study by Meremikwu et al. in 2007, reported laboratory test rate of 45% [8], while Uzochukwu et al. reported a rate of 51.1% in Enugu [21], suggesting no significant improvement since 2010. The level of confidence in clinical diagnosis should be considered unrealistic in view of the evidence to the contrary and the high incidence of inaccuracy and wastages associated with presumptive malaria treatment [22]. Consequences include missed diagnosis of other illnesses and increased risk of morbidity [23]. This underscores the need for intensified efforts at promoting the use of diagnostic approach to malaria treatment at the facilities, through regular education programmes for health workers [3]. In addition, findings also suggest that low utilisation of diagnostic test was due to high patient load and hence, lack of waiting time for receiving the result of the test, which should be noted for improvement. The benefit of RDT in terms of rapid delivery of results addresses this problem. Patients were treated presumptively even with the availability of laboratory tools in the facilities. The proportion of patients who received antimalarial drugs with slide negative results was quite substantial, considering the enormous wastages that accompany this. This was justified by the prescribers for a number of reasons; (1) unreliability of laboratory results due to poor laboratory reagents, (2) RDT insensitivity (further studies may be required to explore this), and 3) unaware of laboratory results. Previous studies in the area have similarly reported unreliability of laboratory results as a major cause of treatment of slide negative results, especially with RDT [10, 21]. Hence, the study showed limited use of RDT compared to microscopy, even when it was available in both facilities, confirming the lack of trust of providers on RDT test results. This should worry policy considering the international focus on the use of RDT and implication for the goal of TTT policy [2, 3]. Further investigation on the supply of quality RDT products for intervention is required, to ensure reliability of its results and the success of the test and treat policy. There is clear need for improved laboratory standards for malaria diagnosis, which can be achieved through a simple system of quality control. Continuous education of providers through regular seminars and workshops, on the benefits of confirmatory diagnosis cannot be over-emphasized. The extent of treatment of slide negative results and doctors responses to the issue calls for strategies to enhance their respect for negative results. There is need to enforce quality control to enhance reliability of diagnostic results. Benefits of confirmatory diagnosis and consequences of poor laboratory practices should be part of the regular updates to boost the confidence of prescribers in adhering to laboratory diagnosis.

Pattern of prescription in the study shows a clear preference for ACT, as the drug of choice for uncomplicated malaria at the two health facilities. This indicates high conformity to policy recommendation. The preference for AL, the policy first line drug at both facilities indicates providers’ confidence in the efficiency of the regimen, as was confirmed by questionnaire responses. The pattern appears similar to what obtained in the retail sector in the area, where DHAPQ was also found to be the second most prescribed antimalarial drug/ACT [11]. However, the greater dominance of ACT in this study, 95% compared to the retail sector 73%, is consistent with findings that public health facilities conform more to policy guidelines than the retail sector [11]. The retail sector is dominated by self-medication, which is characterised by high incidence of monotherapy use. The fact that the medical center uses more ACT than the tertiary health facility can be explained by the predominance of children and female cases at the later in which monotherapy was most prescribed for prophylaxis. Preference for AL is consistent with many study findings in both Nigeria and other African countries [3, 11, 18]. Similar to the retail sector [11], the use of AA which was the policy’s alternative policy drug, was limited in this study. This was also explained by the reported safety concerns associated with the use of AA, especially in adults known to present with varying degrees of side effects [11]. The prescription of SP, mostly for prophylaxis in the study conforms to guidelines for its use in Intermittent Preventive Treatment in pregnancy and children (IPTp and IPTc). However, the use of AS and quinine is not in line with policy and therefore should be of concern.

The use of an average of four drugs per prescription in this study suggests high incidence of co-medication, going by the WHO recommendation of two to three drugs for developing countries. This gives an indication of poly-pharmacy in the studied facilities, increasing the risks of drug interactions, adverse drug reactions and high cost of treatment for the patients. Co-medication was higher at the medical center with five drugs per prescription. While poly-pharmacy may be justified in some cases by the significant number of co-morbidity, proportion of co-medication with vitamin preparations and antibiotics has implications for the safety and efficacy of antimalarial drugs. The fact that many of the prescriptions were on the basis of presumptive diagnosis, made this situation more critical, contributing to further wastages. There are concerns with co-administration of vitamin preparations with ACT in view of the antioxidant effects of vitamin compounds such as zinc, iron, vitamins C and E, on artemisinin compounds [24, 25]. This may lead to reduced availability and hence reduced efficacy of the agents, and in consequence, contribute to treatment failures and increasing resistance of the *Plasmodium*. It has been advised that if needed, vitamins preparations could be used after completing the ACT dose. The use of antibiotics in absence of co-morbidity also has implications for safety, in view of their known side effects, which may be wrongly attributed to the antimalarial drugs. Co-prescription with antibiotics occurs usually as ‘a cover’ for potential co-infection which would suggest that the prescriber is less confident of actual diagnosis, or prevent subclinical infection becoming manifest [26]. Hence, many prescriptions were secondary to diagnosed infections. Similar findings were reported from a study in Tanzania, for patients with a history of cough in the last 48 hours, for which antimalarials were prescribed even with negative results [15].

Although this study did not assess the appropriateness of antibiotics use, the likelihood of overtreatment with antibiotics, similarly reported in many other studies [26, 27] had led to the call for better diagnostic approach to non-malarial fevers and development of guidelines for management of such illnesses, which should be incorporated into malaria case-management trainings for health workers [3]. This recommendation should be treated as priority to enhance the rational use of both antimalarial drugs and antibiotics.

The total cost of medication per prescription (including co-medication) in this study, which showed a median of US$7.48, is about 2.6 times higher than similar cost obtained for retail outlets, (US$2.90) in a study undertaken at about the same period in a neighbouring city [11]. This is consistent with reported higher cost of care in public health facilities, due to the cost of more professional services [9]. This relatively higher cost of medication in public health facilities has remained as one of the major factors that inform the preference for the retail sector by a significant proportion of patients’ population for malaria treatment [9]. The higher cost of medication at the tertiary health facility may be explained by the higher cost of expert care at a tertiary/referral center.

Treatment practices varied notably between the two facilities, in terms of patients’ characteristics. The p-value shows significance in many of the variables, indicating differences in prescribing practices of doctors between the facilities. These differences highlight the variation in prescribing cultures between similar facilities across the country, suggesting differences in dissemination of anti-malaria training information. The differences may also point to the levels of exposure to malaria treatment practices. Regular updates therefore provide opportunity for promoting appropriate malaria treatment practices in these health facilities [18]. Indicators suggest better performances at the teaching hospital compared to the medical center, which is consistent with reports that prescribers in tertiary institutions tend to adhere more to national treatment guidelines [28]. The presence of more specialized doctors at the tertiary center, who are probably better exposed to information than those at the medical center, may explain this.

## Limitations

A few limitations are reported in this study. The selected sample facilities may reflect a potential bias towards public health facilities with high patient load. However, considering the health-seeking pattern for malaria treatment in Nigeria, where majority of cases are treated in the public sector compared to the private sector [29], selected facilities may be a likely representative of study population. Comprehensive diagnostic information was not collected due to inadequate documentation of patient diagnosis, to better inform the use of antibiotics and other concomitant medications. The study did not assess the appropriateness of prescription dosages and weights. However, most of the ACTs were administered according to age-related dose packages and hence most likely to conform to patients’ ages and weights. While the study may be limited in scope in terms of the number of facilities studied, findings reflect, to a greater extent the practice pattern in the sector, considering the similarity in many ways, of the findings of previous studies carried out in the area [7, 8, 10, 21] and other settings [26]. However, the study needs to be scaled-up to strengthen the findings for enhanced policy interventions for improved malaria case management.

## Conclusion

Eight years after the change in antimalarial treatment policy, there is substantial compliance to policy on the use of ACT, as the first line treatment for uncomplicated malaria at the two health facilities. However, treatment practices are substantially characterised by limited use of laboratory diagnosis, relying mostly on presumptive treatment, over-diagnosis, over-treatment, co-medication and lack of routine information on malaria treatment, to guide effective implementation of treatment guidelines. These create the risk of developing parasite resistance and treatment failures, undermining the goals of malaria treatment policy. There is therefore a wide scope for improved diagnostic and treatment practices at the two health facilities, to enhance the efficiency of malaria case management. Targeted intervention through promotion and regular education of providers on appropriate malaria treatment practices is imperative, based on recommended guidelines and the test and treat policy. This would surely improve confirmatory diagnosis and rational drug prescribing, to achieve the goals of malaria case management. There is also the need to ensure adequate supply of quality and sensitive diagnostic equipments, which is critical to the success of the ‘test and treat’ policy of malaria case management.
